# Electric Field Modulation of Interlayer Coupling via Piezostrain in a Synthetic Antiferromagnet

**DOI:** 10.1002/advs.202517798

**Published:** 2025-11-28

**Authors:** Yuichi Hisada, Sachio Komori, Keiichiro Imura, Chenyu Shen, Yoshihiro Gohda, Calvin Ching Ian Ang, Wen Siang Lew, Tomoyasu Taniyama

**Affiliations:** ^1^ Department of Physics Nagoya University Furo‐cho, Chikusa‐ku Nagoya 464‐8602 Japan; ^2^ Department of Materials Science and Engineering Institute of Science Tokyo Yokohama 226‐8501 Japan; ^3^ School of Physical and Mathematical Sciences Nanyang Technological University 21 Nanyang Link Singapore 637371 Singapore

**Keywords:** artificial multiferroic structures, electric field effect, interlayer exchange coupling, spintronics, synthetic antiferromagnets

## Abstract

Controlling the interlayer exchange coupling (IEC) in synthetic antiferromagnets (SAFs) using an electric field is a promising approach for developing energy‐efficient spintronic devices, as it enables magnetization switching without electrical current. In this study, the modulation of the IEC through electric field‐induced strain in a Co/Ru/Co SAF on a Pb(Mg_1/3_Nb_2/3_)O_3_‐PbTiO_3_ (PMN‐PT) multiferroic heterostructure is demonstrated. It is found that both the IEC and the uniaxial magnetic anisotropy energy are modulated by applying an electric field to the Co/Ru/Co/PMN‐PT structure. This modulation is evident from the behavior of the minor hysteresis loops observed in our experiments and micromagnetic simulations. Additionally, it is clarified that the in‐plane piezoelectric strain transferred from the PMN‐PT to the Co/Ru/Co SAF layer enhances the strength of the antiferromagnetic IEC. Notably, the efficiency of this enhancement due to piezoelectric strain is strongly correlated with the thickness of the Ru spacer, a finding that aligns with our first‐principles calculations. Controlling the IEC via the piezoelectric strain transfer effect using an electric field enables the manipulation of antiferromagnetic order with extremely low energy consumption, offering significant potential for energy‐efficient spintronic memory devices.

## Graphical abstract

Controlling the interlayer exchange coupling (IEC) in synthetic antiferromagnets (SAFs) using an electric field is a promising route toward energy‐efficient spintronic devices. In this study, electric field induced‐strain modulation of IEC in Co/Ru/Co SAF/PMN‐PT is demonstrated. The modulation efficiency depends on the initial IEC strength; stronger IECs lead to higher modulation efficiencies.

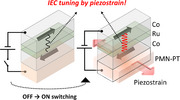

## 1 Introduction

The advent of antiferromagnetic (AFM) spintronics,^[^ ^1^, ^2^ ^]^ which leverages the fascinating features of AFM materials, such as the absence of stray fields, robustness against high magnetic fields, and ultrafast magnetization dynamics in the terahertz (THz) band,^[^ ^3^, ^4^, ^5^, ^6^ ^]^ has made the manipulation of AFM order a subject of numerous investigations for AFM spintronic memory devices. Recent studies have demonstrated that the AFM order in such materials can be manipulated by a spin‐polarized electric current.^[^ ^7^, ^8^, ^9^, ^10^ ^]^ However, controlling the AFM order using low electric current densities remains challenging due to the extremely strong exchange interaction in AFM materials.

A synthetic antiferromagnet (SAF), i.e., a ferromagnetic (FM)/nonmagnetic (NM)/ferromagnetic (FM) multilayer with an AFM interlayer exchange coupling (IEC) between two FM layers, the strength and sign of which depend heavily on the thickness of the NM layer,^[^ ^11^ ^]^ is key to overcoming these challenges.^[^ ^12^ ^]^ Since the IEC strength is much weaker than the exchange interaction strength in AFM materials, the AFM order in SAFs can be controlled with a low electric current density.^[^ ^12^ ^]^ Due to these unique features, SAFs have been intensively studied in the field of spintronics, for example in spin‐polarized current‐driven magnetization reversal for magnetoresistive random‐access memory,^[^ ^13^, ^14^, ^15^ ^]^ sub‐THz excitations for spin‐torque nano‐oscillators,^[^ ^16^, ^17^ ^]^ and domain wall or magnetic skyrmion motion for racetrack memory.^[^ ^18^, ^19^, ^20^ ^]^ Furthermore, recent research has demonstrated voltage control of the IEC in SAFs via magneto‐ionic gating using hydrogen,^[^ ^21^ ^]^ oxygen,^[^ ^22^ ^]^ and solid‐state lithium ions,^[^ ^23^ ^]^ as well as an ionic liquid gating method.^[^ ^24^, ^25^ ^]^ This approach enables 180‐degree magnetization reversal via FM/AFM coupling switching, eliminating the need for high electric current densities. However, although the IEC can be switched without a current through the magneto‐ionic gating method, there are some issues regarding the realization of efficient spintronic devices. These include a lack of long‐term repeatability due to magnetic thin film degradation through ionic infiltration, a time‐consuming change in magnetic property (on a timescale of more than 1 s) and a relatively short propagation length of ionic infiltration into metallic films (∼a few nm).

Another promising method of electric field control of IEC involves using an artificial multiferroic structure composed of a magnetic multilayer and ferroelectrics (FE). These structures exhibit magnetoelastic coupling at the interface between the magnetic multilayer and the FE via piezostrain in an electric field. This can control the magnetic anisotropy and magnetization of magnetic materials.^[^ ^26^, ^27^ ^]^ Moreover, the propagation length of this piezostrain is typically several hundred nanometers into a metallic film, and relatively fast magnetization switching can be achieved via magnetoelastic coupling in a multiferroic heterostructure (≈0.1 µs),^[^ ^28^ ^]^ which has great potential for energy‐efficient spintronic devices. Recently, the effect of piezostrain on the static and dynamic magnetization processes in SAF/FE multiferroic heterostructures has been reported.^[^ ^29^, ^30^, ^31^, ^32^ ^]^ Wang et al. demonstrated that magnetization curves in FeCoB/Ru/FeCoB SAF on Pb(Mg_1_/_3_Nb_2_/_3_)O_3_‐PbTiO_3_ (PMN‐PT) multiferroic heterostructures switch between single‐loop and double‐loop patterns via piezoelectric strain transfer from PMN‐PT to the SAFs.^[^ ^29^ ^]^ Furthermore, a previous study by our group has clarified that the effect of electric field‐induced strain on magnetic anisotropy in polycrystalline Co/Ru/Co SAF on PMN‐PT heterostructures depends strongly on the strength of the IEC.^[^ ^31^ ^]^ As the nature of the IEC is determined by the shape of the Fermi surface in the NM layer,^[^ ^33^, ^34^ ^]^ the distortion of the Fermi surface by strain, such as piezostrain, could modulate the IEC oscillation period and/or strength.^[^ ^35^ ^]^ However, little experimental evidence of IEC modulation through piezostrain has been found in SAFs/FE multiferroic structures,^[^ ^29^, ^30^ ^]^ and the piezostrain response to IEC has also not been sufficiently investigated.

Here, we demonstrate electric field‐induced strain modulation of the IEC in Co/Ru/Co/PMN‐PT epitaxial multiferroic heterostructures. We confirm that the switching magnetic fields of the minor hysteresis loops ‐ a measure of IEC strength ‐ are clearly modulated by the application of an electric field. Micromagnetic simulations based on the Mumax3 package^[^ ^36^, ^37^ ^]^ are performed to model the electric‐field modulation behavior of the minor hysteresis loops obtained in our experiments. We find that the enhancement of the AFM IEC and the reduction of magnetic anisotropy energy determine the magnetization process in Co/Ru/Co SAF heterostructures under electric fields. We reveal that tensile strain in the PMN‐PT (011) plane is crucial for controlling the IEC, and the efficiency of electric field manipulation of the IEC strongly depends on the thickness of the intermediate Ru layer. This is qualitatively consistent with our first‐principles calculations. This method of modulating the IEC using strain has great potential for achieving magnetization switching with extremely low energy consumption, paving the way for energy‐efficient spintronic devices.

## 2 Crystallinity of Co/Ru/Co/PMN‐PT Heterostructures

**Figure 1:**
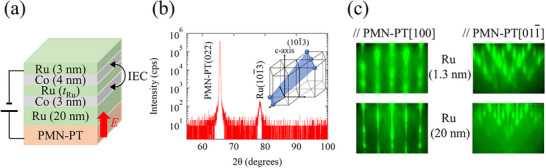
a) A schematic illustration of a Ru (3 nm) /Co (4 nm) /Ru (*t*_Ru_) /Co (3 nm) /Ru (20 nm) /PMN‐PT(011) multiferroic structure. b) Out‐of‐plane XRD pattern for the sample with *t*_Ru_ = 1.3 nm. The inset figure denotes Ru $\left(10\bar{1}3\right)$ plane. c) RHHED patterns after deposition of Ru (20 nm) and Ru (1.3 nm) layers along PMN‐PT[100] and PMN‐PT $\left[01\bar{1}\right]$ directions.

**Figure** **1**a shows a schematic illustration of a multiferroic heterostructure consisting of Ru (3 nm)/Co (4 nm)/Ru (*t*_Ru_)/Co (3 nm)/Ru (20 nm) and PMN‐PT(011), where *t*_Ru_ is the thickness of the Ru spacer layer. X‐ray diffraction (XRD) measurements were carried out to determine the crystallinity of our samples. We find that the Ru layer(s) are oriented along the $\left(10\bar{1}3\right)$ plane, as shown in Figure 1b. As the Ru buffer layer is thicker than the other Ru layers, the Bragg diffraction peak is predominantly attributed to the buffer layer. To confirm the crystallinity of the other Ru spacer layers, especially the 1.3‐nm Ru layer, Figure 1c shows in situ reflection high energy electron diffraction (RHEED) patterns of the 20 and 1.3‐nm Ru layers. Although the streaky pattern of the 1.3‐nm Ru layer is broader than that of the 20‐nm Ru layer, the two diffraction patterns are almost identical, indicating that the 1.3‐nm Ru layer is likely oriented along the $\left(10\bar{1}3\right)$ plane. The Bragg diffraction peak from the Co layer is undetectable, which suggests that the grain size of the Co thin films is small. For the first‐principles calculation of the strain effect on the IEC in Co/Ru/Co SAFs, which will be discussed later, we assume that the Co layers are oriented along the $\left(20\bar{2}1\right)$ plane due to the small lattice mismatch with the Ru $\left(10\bar{1}3\right)$ plane.

## 3 Ru‐Thickness Dependent IEC Strength

**Figure 2:**
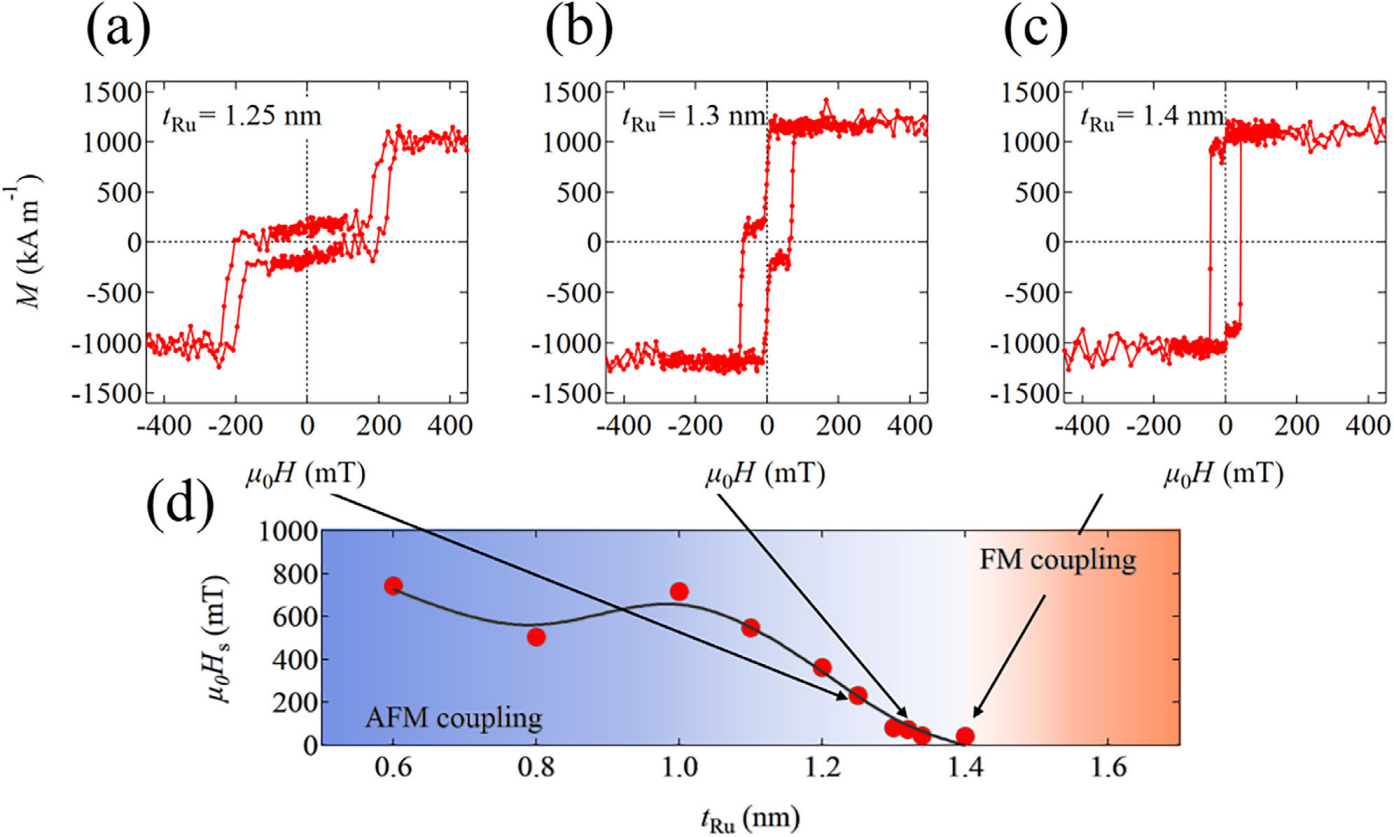
a–c) Magnetization hysteresis curves (*M*–*H* curves) along PMN‐PT $\left[01\bar{1}\right]$ for the samples with *t*_Ru_ = 1.25, 1.3, and 1.4 nm, respectively. d) Magnetic saturation field (*µ*_0_ *H*_s_) as a function of *t*_Ru_. The black curve is a guide to the eye.

**Figure** **2**a–c show the magnetization hysteresis curves (*M*‐*H* curves) along the $\mathrm{PMN}-\mathrm{PT}[01\bar{1}]$ direction (i.e., the easy axis) for samples with *t*_Ru_ = 1.25, 1.3, and 1.4 nm, respectively. Figure 2d shows the saturation magnetic field (*µ*_0_ *H*_s_) as a function of *t*_Ru_. In general, *µ*_0_ *H*_s_ is closely associated with IEC strength in SAFs.^[^ ^38^, ^39^ ^]^ Therefore, it is evident that the IEC strength exhibits oscillatory behavior as a function of *t*_Ru_. In the sample with *t*_Ru_ = 1.4 nm, the plateau where the magnetizations of the two Co layers are antiparallel disappears, suggesting that the sample exhibits FM coupling. This suggests that the boundary between AFM and FM coupling is around *t*_Ru_ = 1.4 nm, consistent with previous studies of epitaxially grown Co/Ru/Co SAFs.^[^ ^40^, ^41^ ^]^ Notably, the metamagnetic transition is clearly visible in samples with AFM coupling, indicating that the strength of the uniaxial magnetic anisotropy (*K*_u1_) is much greater than that of the IEC constant *J* (i.e., *K*_u1_ >> −*J*/*t*, where *t* is the thickness of the two FM layers).^[^ ^38^, ^39^ ^]^ The *K*_u1_ value of the sample with *t*_Ru_ = 1.3 nm is determined from the saturation magnetic field measured along magnetic hard axis (see Figure S1, Supporting Information) and found to be approximately one order of magnitude larger than the *J*/*t* value (see Note S1, Supporting Information).

## 4 Electric Field Effects on the IEC

**Figure 3:**
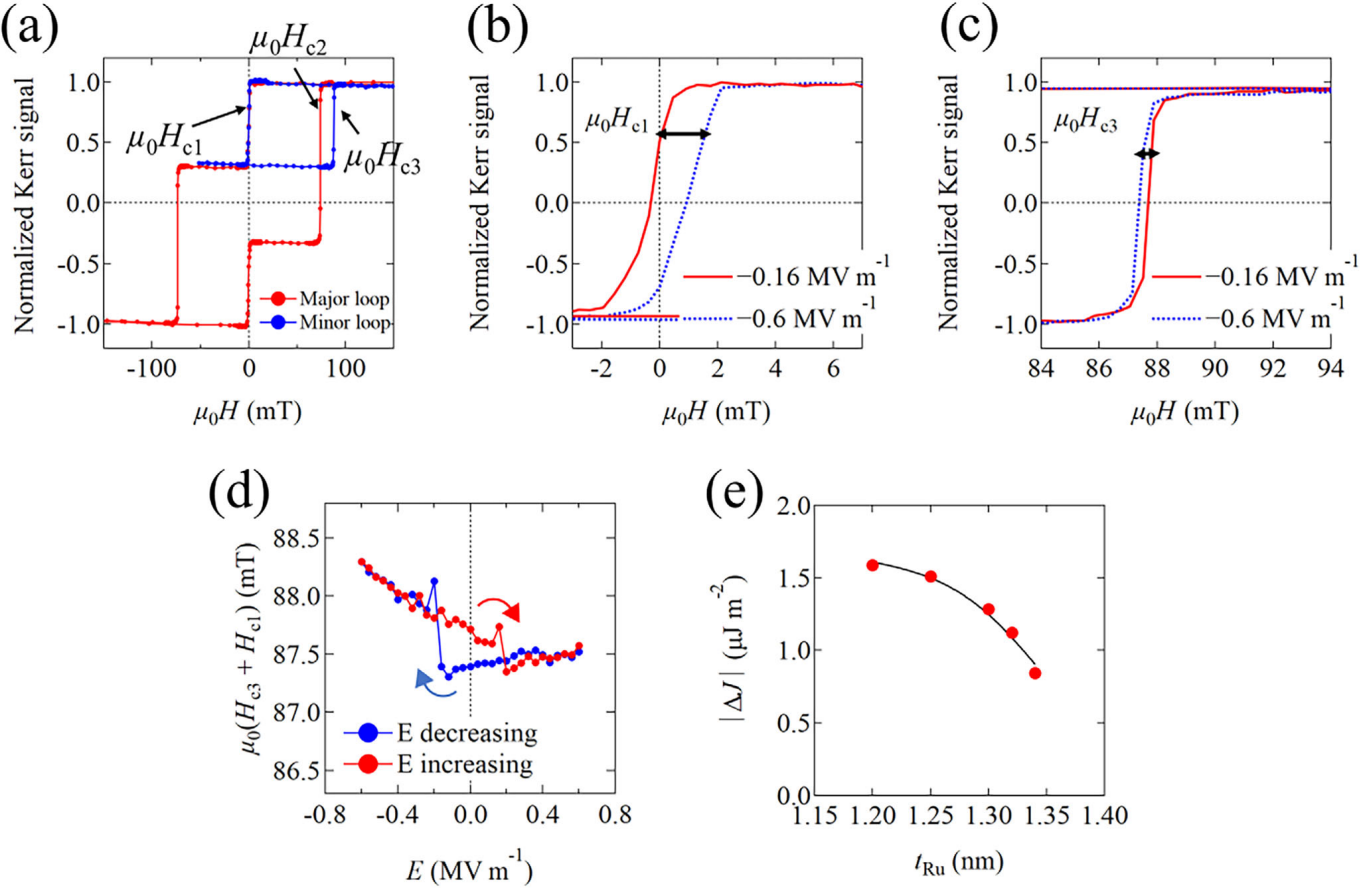
a) A typical major and minor hysteresis curve. *µ*_0_ *H*_c1_, *µ*_0_ *H*_c2_, and *µ*_0_ *H*_c3_ are the magnetic switching field at which each Co layer exhibits independent magnetization switching as shown in the inset of Figure 3a. (b,c) Minor hysteresis loops under *E* = −0.16 (red line) and −0.6 MV m^−1^ (blue dot line) near the *µ*_0_ *H*_c1_ and *µ*_0_ *H*_c3_, respectively. (d) *µ*_0_(*H*_c1_ + *H*_c3_) as a function of *E*. Blue (red) line denotes the data swept from *E* = +0.6 MV m^−1^ (−0.6 MV m^−1^) to *E* = −0.6 MV m^−1^ (+0.6 MV m^−1^). e) Ru layer‐thickness dependence of the amount of change in *J* before and after electric polarization reversal in PMN‐PT.

Although the IEC constant *J* is characterized by *µ*_0_ *H*_s_, they are not proportional due to the much larger value of *K*_u1_ compared to *J*.^[^ ^38^, ^39^ ^]^ Therefore, to determine the exact value of *J*, minor hysteresis loops were measured under electric fields. **Figure** **3**a shows the typical major and minor hysteresis loops of our Co/Ru/Co SAF, as measured by a magneto‐optical Kerr effect (MOKE) magnetometer. The magnetization switching fields (*µ*_0_ *H*_c1_, *µ*_0_ *H*_c2_, *µ*_0_ *H*_c3_) in the major and minor hysteresis loops are defined as shown in Figure 3a. The *µ*_0_(*H*_c1_ + *H*_c3_) value is proportional to the IEC constant *J* ^[^ ^42^ ^]^ and the effect of the electric field on the IEC can be estimated from the minor hysteresis loops. Figure 3b,c show minor hysteresis loops under electric fields (*E*) of −0.16 MV m^−1^ (red curve) and −0.6 MV m^−1^ (blue curve), respectively, for a sample with *t*_Ru_ = 1.3 nm, in the vicinity of *µ*_0_ *H*_c1_ and *µ*_0_ *H*_c3_. The switching magnetic fields of *µ*_0_ *H*_c1_ and *µ*_0_ *H*_c3_ are clearly modulated by the application of an electric field. We plot the *µ*_0_(*H*_c1_ + *H*_c3_) value, i.e., the IEC strength, against *E*, as shown in Figure 3d. Notably, clear hysteresis‐like modulation behavior of the AFM IEC strength with respect to the electric field can be observed, demonstrating the electric field control of the IEC in SAFs/FE multiferroic heterostructures. However, the IEC constant against *E* is different for all samples, as shown in Figure S2 (Supporting Information): a butterfly‐like behavior versus *E* is observed for the sample with *t*_Ru_ < 1.25 nm, while hysteresis‐like behavior is observed for the sample with *t*_Ru_ > 1.3 nm. This may be due to the different ferroelectric properties of each PMN‐PT substrate, which will be discussed in Section 5. As shown in Figure 3d, the *µ*_0_(*H*_c1_ + *H*_c3_) value increases (decreases) sharply at *E* = −0.16 (MV m^−1^) [+0.16 (MV m^−1^)] as *E* decreases (increases). This *E* value corresponds to the electric field at which the electric polarization reverses in the PMN‐PT substrates. This suggests that an inverse piezoelectric strain transfer from the PMN‐PT to the SAFs leads to an enhancement or reduction in the AFM coupling strength, since the PMN‐PT generally exhibits a large in‐plane strain when the polarization vector switches.

To investigate the effect of the electric field on the IEC in detail, Figure 3e shows the absolute value of Δ*J* (|Δ*J*|) as a function of *t*_Ru_, where Δ*J* is defined as the amount of change in *J* before and after electric polarization reversal in PMN‐PT. We use Equation S6 (Supporting Information) to extract *J* from *µ*_0_(*H*_c1_ + *H*_c3_) (see Note S2, Supporting Information). It can be seen that |Δ*J*| decreases as *t*_Ru_ increases. This indicates that the IEC strength of SAFs with strong AFM coupling can be modulated more efficiently by applying an electric field than SAFs with weak coupling. This trend is qualitatively consistent with our first‐principles calculations, which will be discussed later.

## 5 Piezoelectric Strain in PMN‐PT(011)

**Figure 4:**
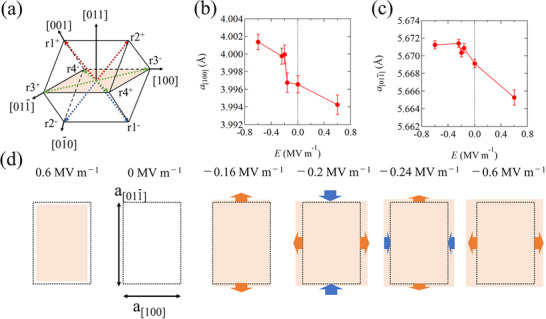
a) A schematic illustration of PMN‐PT crystal structure with spontaneous polarization vectors along the <111> directions. The colored square represents (011) plane. b,c) show the electric field dependent lattice parameter of PMN‐PT along [100] and $\left[01\bar{1}\right]$, respectively. Electric fields were swept from +0.6 to −0.6 MV m^−1^. d) Schematic illustrations of the in‐plane strain behavior of PMN‐PT during electric field sweeping.

We investigate the in‐plane strain behavior of the PMN‐PT substrate in order to identify the origin of the electric field modulation of IEC. **Figure** **4**a shows a schematic illustration of the PMN‐PT(011) crystal structure with eight spontaneous electric polarization vectors along the <111> direction. Under an electric field, these electric polarization vectors switch through pathways including 71° (r1+ → r3+/r4‐) and 109° (r1+ → r4+/r3‐) ferroelastic domain switching and 180° (r1+ → r1‐) ferroelectric domain switching. To investigate the in‐plane strain induced by ferroelastic domain switching in detail, in‐plane XRD measurements were performed around the diffraction spot of the PMN‐PT substrate under electric fields. Figure 4b,c shows the lattice parameters along the PMN‐PT [100] (*a*_[100]_) and $\left[01\bar{1}\right]$ ($a_{\left[01\bar{1}\right]}$) directions as a function of applied electric field, obtained from the diffraction patterns of the PMN‐PT (100) and $\left(01\bar{1}\right)$ planes for the sample with *t*_Ru_ = 1.3 nm, respectively. The electric field is swept from +0.6 to −0.6 MV m^−1^, corresponding to the field sweep sequence of the blue curve in Figure 3d. Each diffraction pattern is fitted with a Gaussian function, as shown in Figure S3 (Supporting Information). We find that the lattice constant for both *a*_[100]_ and $a_{\left[01\bar{1}\right]}$ increases (decreases) when a negative (positive) electric field is applied, suggesting an asymmetric modulation of the lattice constant with respect to the polarity of the applied electric field. It can also be seen that the lattice constant, particularly for *a*_[100]_, changes drastically at *E* = −0.16 to −0.2 MV m^−1^, a value that corresponds to the electric polarization reversal of PMN‐PT. These responses to electric fields are well compatible with the IEC modulation behavior, as shown in Figure 3d. This indicates that piezoelectric strain transfer from PMN‐PT to SAFs is crucial for controlling the IEC. The in‐plane strain behaviors at each electric field value are summarized in Figure 4d. The dots and colored squares represent the PMN‐PT(011) plane at zero and non‐zero electric fields. At *E* = −0.16 MV m^−1^ (just before the electric polarization reversal), a tensile strain occurs in the [$01\bar{1}$] direction, with almost no strain in the [100] direction. When an electric field of −0.2 MV m^−1^ is applied, a large tensile (compressive) strain arises along the [100] ($\left[01\bar{1}\right]$) direction. After the electric polarization reversal, the strain in the [100] direction increases further as the negative electric field increases, while the strain in the $\left[01\bar{1}\right]$ direction exhibits saturation behavior until *E* = −0.6 MV m^−1^. Furthermore, the strain in the [100] direction is approximately a factor of 9 greater than in the $\left[01\bar{1}\right]$ direction from *E* = −0.16 to −0.2 MV m^−1^ (0.08% in the [100] direction and 0.009% in the $\left[01\bar{1}\right]$ direction). We also evaluate the strain in the sample with *t*_Ru_ = 1.2 nm (see Figure S4, Supporting Information). Intriguingly, a large tensile strain in the direction of [$01\bar{1}$], not [100], is observed after polarization reversal in PMN‐PT. Moreover, the electric field dependence of the strain along the [$01\bar{1}$] aligns well with the IEC response to the electric field, while that along [100] is qualitatively consistent with the IEC response in the sample with *t*_Ru_ = 1.3 nm. In other words, the predominant direction of strain for modulating the IEC differs by 90°. Although PMN‐PT substrates we used exhibit complicated strain behavior in each sample, these results imply that the tensile strain is the dominant factor in AFM IEC enhancement.

Here, we discuss the complicated strain behavior in the PMN‐PT substrates of each sample. It should be noted that, while PMN‐PT(011) exhibits complicated strain behavior in the (011) plane around the electric polarization reversal, tensile (compressive) strain generally arises in the $\left[01\bar{1}\right]$ (or [100]) direction as the positive/negative electric field increases further. This strain behavior can be explained by considering the polarization rotation in the rhombohedral crystal structure, as follows.^[^ ^43^ ^]^ When a positive (negative) electric field greater than the coercive electric field is applied, the polarization vectors switch toward r1^+^/r2^+^ (r1^−^/r2^−^) and the polarization vectors projected onto the (011) plane lie along the [100] direction. This generates a tensile strain along the [100] direction and a compressive strain along the $\left[01\bar{1}\right]$ direction. As the electric field increases further, the polarization vectors gradually rotate toward the [011] ($\left[0\bar{1}\bar{1}\right]$) direction, with the [011] axis acting as the rotational axis. This process induces compressive (tensile) strain along the [100] ($\left[01\bar{1}\right]$) direction due to the polarization projected onto the (011) plane lying along the $\left[01\bar{1}\right]$ direction. The above discussion is consistent with the strain behavior in the sample with *t*_Ru_ = 1.2 nm (Figure S4, Supporting Information). However, the sample with *t*_Ru_ = 1.3 nm exhibits tensile strain along the [100] direction under a large negative electric field of −0.6 MV m^−1^, which is inconsistent with the above discussion. One possible reason is that an electric field of −0.6 MV m^−1^ is insufficient for saturating the polarization along the [011] direction, likely due to the asymmetric behavior of polarization versus electric field caused by the imprint effect,^[^ ^44^ ^]^ the flexoelectric effect,^[^ ^45^ ^]^ etc. Since the PMN‐PT(011) substrate we use is in the vicinity of the morphotropic phase boundary (MPB), a structural phase transition induced by applying an electric field should also be considered. Some research has demonstrated that [011]‐poled PMN‐PT near the MPB exhibits a structural phase transition in the rhombohedral‐monoclinic‐orthorhombic phase sequence as the electric field increases,^[^ ^46^, ^47^ ^]^ which could also induce a unique in‐plane strain similar to that observed in our samples. Therefore, we conclude that this structural phase transition is one of the key factors for determining the distinct behavior between butterfly‐like or hysteresis‐like IEC responses (see Figure S5 in Note S6, Supporting Information). Since this study mainly focuses on the response of the IEC in SAFs to electric field‐induced strain, rather than the origin of the ferroelectric property under an electric field, investigating the intrinsic and/or extrinsic ferroelectric properties in more detail, such as the imprint effect, the flexoelectric effect and the phase transition in PMN‐PT, is beyond the scope of this study.

## 6 Micromagnetic Simulation for Minor Hysteresis Loop

**Figure 5:**
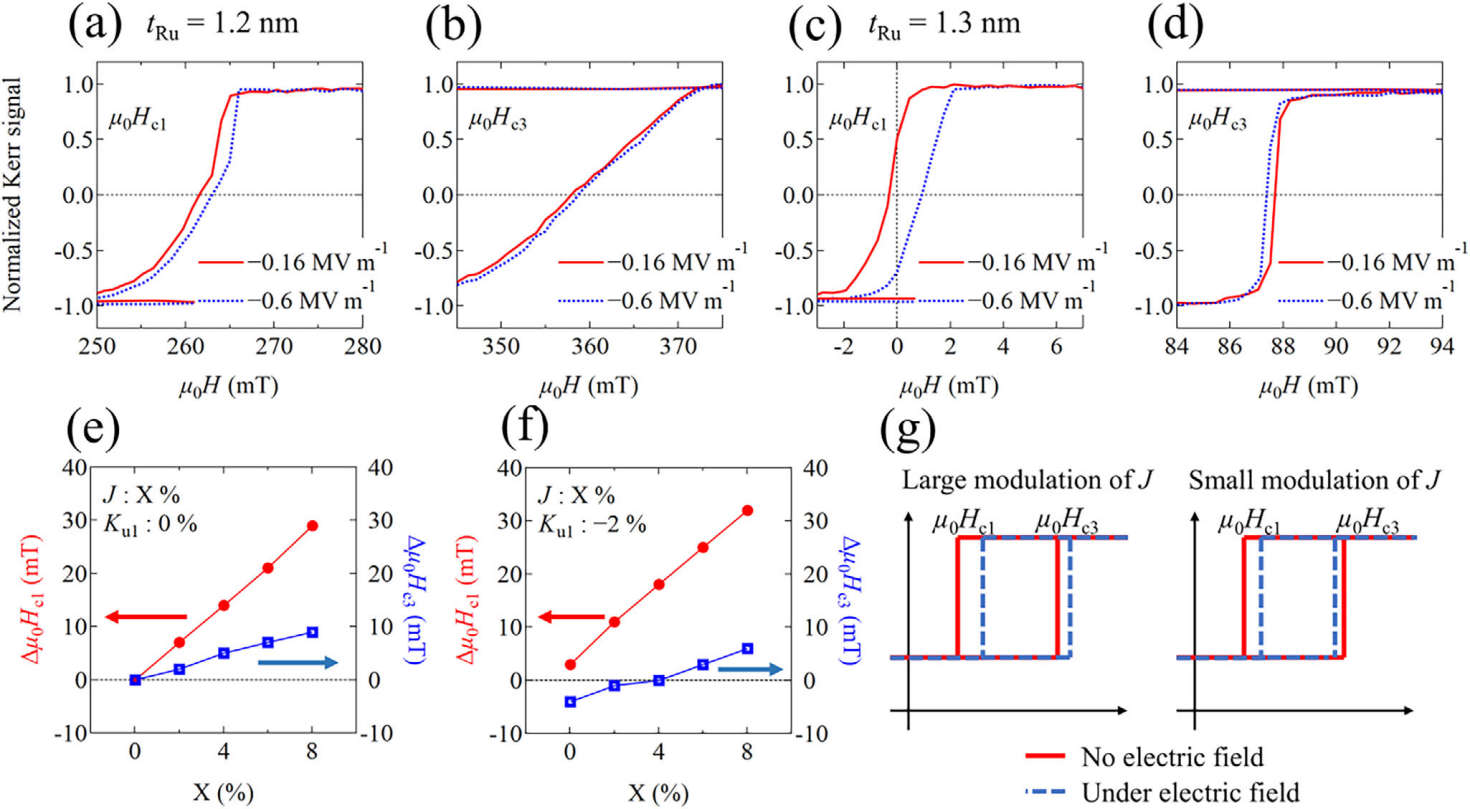
Experimental results of minor hysteresis loops under *E* = −0.16 and −0.6 MV m^−1^ in the vicinity of *µ*_0_ *H*_c1_ and *µ*_0_ *H*_c3_ for samples with *t*_Ru_ = 1.2 nm(a,b) and *t*_Ru_ = 1.3 nm (c,d), respectively. (e,f) Micromagnetic simulation results of the changes in *µ*_0_ *H*_c1_ and *µ*_0_ *H*_c3_ from the initial state (Δ*µ*_0_ *H*_c1_ and Δ*µ*_0_ *H*_c3_) as a function of X (%), where X represents the ratio of the change in *J* from the initial state. Figure 5(e,f) correspond to the situation where the uniaxial magnetic anisotropy constant *K*_u1_ is decreased by 0% and 2%, respectively. g) Schematic illustrations of the minor hysteresis loops under electric fields estimated from micromagnetic simulation results.

**Figure** **5**a–d show the minor hysteresis loops for samples with *t*_Ru_ = 1.2  and 1.3 nm, under *E* = −0.16 and −0.6 MV m^−1^, respectively, near *µ*_0_ *H*_c1_ and *µ*_0_ *H*_c3_. Notably, *µ*_0_ *H*_c1_ and *µ*_0_ *H*_c3_ exhibit different modulation behaviors under electric fields. For the sample with *t*_Ru_ = 1.2 nm, both *µ*_0_ *H*_c1_ and *µ*_0_ *H*_c3_ shift toward higher magnetic fields when an electric field is applied, with *µ*_0_ *H*_c1_ changing much more than *µ*_0_ *H*_c3_. For the sample with *t*_Ru_ = 1.3 nm, however, *µ*_0_ *H*_c3_ decreases while *µ*_0_ *H*_c1_ increases. These results suggest that IEC strength determines the electric field modulation behavior of minor hysteresis loops. The modulation behavior of all our samples can be seen in Figure S6 (Supporting Information). To further understand the differences, micromagnetic simulations are performed to evaluate minor hysteresis loops under various conditions. We simulated the minor hysteresis loops under various values of *K*_u1_ (decreased by 0% and 2%) and *J* (increased toward enhancing the AFM coupling by X%). The detailed conditions in our simulations are described in the Experimental section. Figure 5e,f shows the Δ*µ*_0_ *H*_c1_ and Δ*µ*_0_ *H*_c3_ as a function of X with the various strength of *K*_u1_, where Δ*µ*_0_ *H*_c1_, Δ*µ*_0_ *H*_c3_ are the amounts of change from the initial state, respectively. First, we only change the value of *J* (with no change to *K*_u1_). As shown in Figure 5e, both *µ*_0_ *H*_c1_ (red line) and *µ*_0_ *H*_c3_ (blue line) increase with a positive value as a function of X, indicating that *µ*_0_ *H*_c1_ and *µ*_0_ *H*_c3_ both shift toward a high magnetic field. This result is consistent with the data for the sample with *t*_Ru_ = 1.2 nm (see Figure 5a,b), but not for the sample with *t*_Ru_ = 1.3 nm (see Figure 5c,d), suggesting that other magnetic parameters are also modulated. It should be noted that uniaxial magnetic anisotropy can be modulated by piezoelectric strain in multiferroic heterostructures,^[^ ^26^, ^27^ ^]^ so we should also consider the possibility of magnetic anisotropy modulation in our samples. Figure 5f shows Δ*µ*_0_ *H*_c1_ and Δ*µ*_0_ *H*_c3_ as a function of X when the value of *K*_u1_ is decreased by 2%. Notably, while Δ*µ*_0_ *H*_c1_ (red line) remains positive as in the previous case, the sign of Δ*µ*_0_ *H*_c3_ (blue line) changes from negative to positive as X increases. This indicates that *µ*_0_ *H*_c1_ and *µ*_0_ *H*_c3_ both shift toward the high magnetic field region when *J* undergoes large modulation (high X value), but change in opposite directions when *J* undergoes small modulation (low X value), as shown in Figure 5g. Therefore, we clarify that the magnetization process in the minor hysteresis loop under electric fields can be explained by considering the simultaneous modulation of the IEC and magnetic anisotropy energy. This is based on the fact that the IEC constant *J* for the sample with *t*_Ru_ = 1.2 nm is much larger than for *t*_Ru_ = 1.3 nm, as shown in Figure 3e. We experimentally evaluate the effect of the electric field on uniaxial magnetic anisotropy in minor hysteresis loops and observe a reduction in the magnetic anisotropy energy of the Co (3 nm) layer in almost all samples via the electric field (see Figures S7 and S8, Supporting Information). This is consistent with the qualitative result of the micromagnetic simulation. Additionally, we simulate a minor hysteresis loop by decreasing the *K*_u1_ value while keeping the *J* value, as shown in Figure S9 (Supporting Information). It is confirmed that *K*_u1_ modulation alone cannot explain the electric field‐induced shift in the switching magnetic field obtained in our experiments. Therefore, these simulation results provide solid evidence for the modulation of the IEC in Co/Ru/Co SAFs by an electric field.

## 7 First‐Principles Calculation for Co/Ru/Co System

For the modeling of Co based on $\left(10\bar{1}3\right)$ Ru substrate, as previously discussed, we assume $\left(20\bar{2}1\right)$ plane Co to minimize the lattice mismatch. The in‐plane lattice vectors were identified as *a*_1_ and *a*_2_ via crystallographic transformation (see Figure S10a, Supporting Information). The lattice mismatches *δ*_1_ and *δ*_2_ were evaluated along each direction using

(1) $$\delta_{i}=\frac{a_{i,\;\mathrm{Ru}}-a_{i,\;\mathrm{Co}}}{a_{i,\;\mathrm{Ru}}}\times 100\%$$

**Figure 6:**
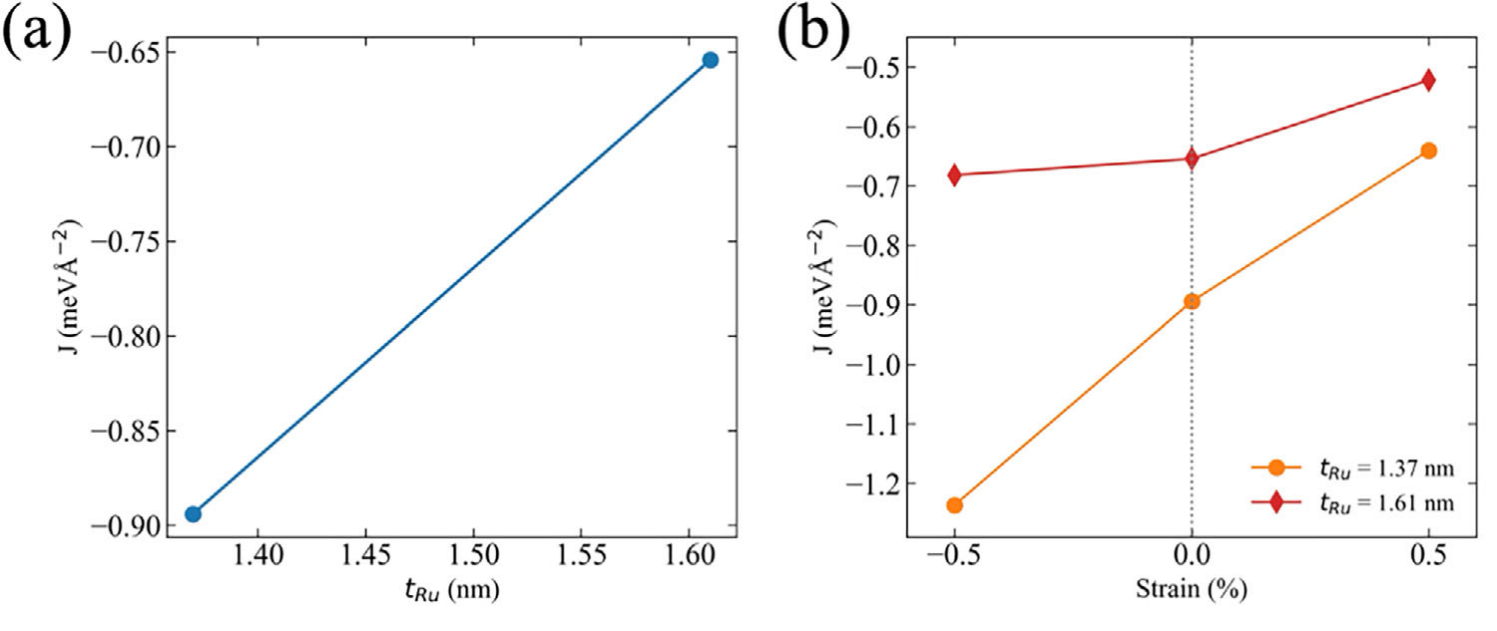
Dependence of exchange coupling constant *J* on Ru thickness and strain. a) Variation of *J* with Ru thickness *t*_Ru_ at zero strain. The magnitude of *J* decreases with increasing *t*_Ru_, indicating weakened exchange coupling in thicker Ru layers. b) Strain response of *J* for different *t*_Ru_ values. Strain sensitivity decreases with increasing *t*_Ru_ from 1.37 to 1.61 nm.

We find mismatches of 8.4% and −0.5% along *a*_1_ and *a*_2_, respectively. To model the experimental structure, 30‐monolayer (ML) and 40‐ML Co are prepared to simulate 3 and 4‐nm Co, respectively. Furthermore, 11 and 13 ML of Ru, corresponding to the Ru thickness *t*_Ru_ of ≈1.37  and 1.61 nm, respectively, are prepared for the calculation (see Figure S10b, Supporting Information). **Figure** **6**a shows calculated IECs for relaxed stable structures with the different *t*_Ru_ values. As *t*_Ru_ increases, the AFM coupling shown as negative IEC values becomes weaker. This tendency of the IEC magnitude reduction aligns consistently with experimental observations shown in Figure 2d. As noted previously, the PMN‐PT(011) substrate shows a complex strain pattern that has anisotropy in [100] and $\left[01\bar{1}\right]$ directions. Given the absence of direct experimental quantification of the specific lattice matching or domain alignment at the PMN‐PT/Ru interface, applying a biaxial strain to the Ru layer is a well‐established and computationally tractable approach for modeling strain effects in Co/Ru/Co heterostructure. This approach enables quantitative calculation of IEC variations under biaxial strain, with results summarized in Figure 6b. As shown in Figure 6b, both tensile and compressive biaxial strains exhibit a monotonic attenuation in the variation of IEC due to strain |Δ*J*| with increasing *t*_Ru_. This thickness‐dependent reduction as a first‐order trend quantitatively aligns with experimental measurements shown in Figure 3e and confirms Ru thickness as a critical IEC tuning parameter. Interfacial thermodynamic stability was further quantified through formation energy per unit area:

(2) $$E_{\mathrm{form}}=\left[E_{\mathrm{total}}-n_{\mathrm{Co}}*\mu_{\mathrm{Co}}-n_{\mathrm{Ru}}*\mu_{\mathrm{Ru}}\right]/A$$

where *E*_total_ denotes the total energy of the Co/Ru/Co heterostructure, *n*_Co_ and *n*_Ru_ are the number of atoms in the supercell, while *µ*_Co_ and *µ*_Ru_ represent the chemical potentials of an atom in bulk hcp Co and hcp Ru, respectively. The normalized formation energy decreases from 4.31 eV Å^−2^ at *t*_Ru_ = 1.37 nm to 3.47 eV Å^−2^ at *t*_Ru_ = 1.61 nm, confirming enhanced thermodynamic stability at greater thicknesses.

## 8 Discussion

Here, we propose a possible mechanism for stain‐induced IEC modulation in SAFs. *J* can be described by the following equation, which is based on quantum interference theory^[^ ^33^, ^34^ ^]^:

(3) JtNM=∑αℏv⊥ακα4π2tNM2ImΔrAαΔrBαeiq⊥αtNMeiχα

where *t*_NM_ is the thickness of the NM layer, $v_{⊥}^{\alpha }$ is the effective Fermi velocity in the interface direction, *κ* ^α^ is the radius of the curvature in the Fermi surface, $\Delta r_{A(B)}^{\alpha }$ is the difference of the reflection amplitude between spin up and down at each interface, *χ* ^α^ is a phase, and $q_{⊥}^{\alpha }$ is a critical spanning vector that is a wavenumber vector connecting the stationary points of two different Fermi surfaces in NM layer. Since multiple critical spanning vectors are present in most NM metals, such as Cu, Au, Ag and Ru,^[^ ^34^, ^48^ ^]^ the components on the right‐hand side of Equation (3) are labelled by α, corresponding to each critical spanning vector. The oscillation period of the IEC, *Λ* ^α^, can be obtained using Equation (4):

(4) $$\Lambda^{\alpha }=\frac{2\pi }{q_{⊥}^{\alpha }}$$

As the critical spanning vector is closely related to the shape of the NM layer's Fermi surface, strain‐induced modulation of the Fermi surface could result in changes to the oscillation period. Previous reports have studied the effect of strain on the Fermi surface of noble metals, both experimentally and theoretically.^[^ ^49^ ^]^ These studies reveal that introducing certain types of strain can modulate the “neck” and other extremal cross‐sectional areas of Fermi surface in noble metals. For example, Fawcett et al. have demonstrated that applying isotropic volume strain to Cu modifies the cross‐sectional area of the “belly” and “neck” orbits,^[^ ^50^ ^]^ which are stationary points related to the critical spanning vector along the [001] and [111] directions of Cu.^[^ ^51^ ^]^

Another possible mechanism is the modulation of the amplitude of spin‐dependent reflection at the interfaces between Co and Ru, inducing a change in the amplitude of *J* (see Equation (3)). In our study, strain transfer from PMN‐PT to SAFs may modify the roughness and/or band structure at the Co/Ru interface(s). This is expected to modify spin scattering behavior at the interfaces and enhance the oscillation amplitude of the IEC via piezoelectric strain transfer.

Note that temperature dependence of the piezostrain effect on the IEC should be considered since both the IEC oscillations and ferroelectric properties are highly sensitive to temperature. In general, IEC becomes stronger as temperature decreases in accordance with *cT*/sinh(*cT*) where *c* is a constant, and *T* is the temperature.^[^ ^33^, ^34^ ^]^ In our study, we clarified that the stronger the IEC is, the more efficiently we can control the IEC via piezostrain. Following this trend, a larger tunability of IEC by piezostrain is expected at low temperature. However, ferroelectric strain responses in PMN‐PT monotonically decrease with temperature.^[^ ^52^ ^]^ Therefore, the balance between the temperature dependence of IEC in the SAFs and piezostrain in PMN‐PT is crucial for controlling the IEC at low temperature.

Further studies are needed to fully understand the mechanism(s) of piezostrain IEC modulation in SAFs, but we believe that introducing an electric field‐induced strain to SAFs is an excellent way to control the IEC, leading to promising candidates for the next generation of spintronic devices.

## 9 Conclusion

In conclusion, we have demonstrated electric field modulation of the IEC in Co/Ru/Co SAF/PMN‐PT multiferroic heterostructures. The magnetic switching fields (*µ*_0_ *H*_c1_ and *µ*_0_ *H*_c3_), which are related to *J* from the minor hysteresis loop, are clearly modulated by the application of an electric field. Micromagnetic simulations have been performed to explain the behavior of the experimental minor hysteresis loops under electric fields. Changing both parameters *J* and *K*_u1_ produces simulated results that agree well with the experimental results. This suggests that applying an electric field to Co/Ru/Co/PMN‐PT heterostructures modulates both the IEC and the uniaxial magnetic anisotropy. In‐plane XRD measurements under electric fields reveal that tensile strain is crucial for enhancing the AFM IEC strength. This in‐plane strain modulation behavior of the IEC is consistent with our first‐principles calculations. The strain‐induced modification of the Fermi surface in the Ru layer and/or at the interfaces between the Co and Ru layers could be a key factor in controlling the IEC in SAFs. This strain‐induced modulation of the IEC in SAFs enables magnetization switching to occur without the need for an electric current, offering the potential for energy‐efficient spintronic memory devices based on SAFs.

## 10 Experimental Section

Co (4 nm)/Ru (*t*_Ru_)/Co (3 nm) SAFs on (011)‐oriented PMN‐PT substrates (PT content *x* = 0.3, i.e., near the MPB, which exhibits a large piezoelectric strain^[^ ^53^ ^]^) were grown by molecular beam epitaxy in an ultrahigh vacuum chamber with a base pressure of better than 1 × 10^−9^ Torr. The thickness of the Ru layer (*t*_Ru_) varied from 0.6 to 1.4 nm. The (011)‐oriented PMN‐PT substrate was selected as the FE material due to its high piezoelectric constant^[^ ^54^ ^]^ and thus FM/PMN‐PT(011) multiferroic structures exhibit a large magnetoelastic effect via piezostrain.^[^ ^55^ ^]^ The PMN‐PT substrates were pre‐annealed at 550 °C for 1 h, followed by the growth of a 20 nm‐thick Ru buffer layer at the same temperature. Subsequently, the SAFs were grown at room temperature. After SAF deposition, a 3 nm‐thick Ru cap was deposited to prevent oxidation of the Co layers. Ti (5 nm)/Au (50 nm) was deposited on the bottom of the PMN‐PT substrates to serve as a bottom electrode, with the SAFs serving as a top electrode. An electric field was then applied along the [011] direction of the PMN‐PT substrates. RHEED measurements were conducted during growth to observe the surface quality and crystallinity of the samples. To confirm the oscillatory behavior of the IEC, *M*‐*H* curves were measured for the samples with various Ru thickness using a vibrating sample magnetometer. The effect of the electric field on the IEC was evaluated using a MOKE magnetometer with a light wavelength of 658 nm. All measurements were carried out at room temperature.

In the micromagnetic simulations, the simulated sample is a Co (3 nm)/Ru (1 nm)/Co (4 nm) trilayer with a saturation magnetization of 1400 kA m^−1^, a Gilbert damping constant of 0.005 and an exchange stiffness constant of 13 pJ m^−1^. It has the shape of a cuboid with dimensions of 256 nm × 256 nm × 8 nm and periodic boundary conditions are set in the sample plane. The initial state is set with *K*_u1_ = 120 kJ m^−3^ and *J* = −0.8 mJ m^−2^. It should be noted that quasi‐static loading and no explicit elasto‐dynamics for the simulation were assumed. In the experiments, the electric field was applied and held for a sufficient duration before measurements were taken, ensuring that the piezoelectric strain had stabilized. Therefore, the quasi‐static loading assumption and the neglect of elasto‐dynamic effects are justified under the measurement conditions. Laterally uniform strain across the magnetic stack and no depth‐dependent strain gradient were also assumed. The strain‐induced changes in *K*_u1_ and *J* were constrained to small strain values, where the magneto‐elastic energy can be effectively mapped to Δ*K*_u1_ and Δ*J*. This is based on the fact that the efficiency of the in‐plane strain modulation of the lattice constant is ≈0.1% as shown in Figure 4b, which should be reasonable for the assumption of the first‐order approximation of magnetoelastic effect in the study.^[^ ^56^ ^]^

First‐principles calculations were performed using the Vienna Ab initio Simulation Package (VASP)^[^ ^57^ ^]^ based on density functional theory (DFT). Projector‐augmented wave (PAW) potentials^[^ ^58^, ^59^ ^]^ with the Perdew–Burke–Ernzerhof (PBE) generalized gradient approximation (GGA)^[^ ^60^ ^]^ were employed. For Co atoms and Ru atoms, a plane‐wave energy cutoff of 348 eV was used for structural relaxation, while 268 eV was used for static calculation. The Co/Ru/Co heterostructure was modeled as a periodic supercell to replicate the experimental structure presented in Section 2. A vacuum layer of ≈15 Å along the *z*‐axis prevented inter‐slab interactions. Biaxial in‐plane strain (*ε*_xx_ = *ε*_yy_ = −0.5% to 0.5%) was imposed while allowing out‐of‐plane relaxation. Atomic positions were optimized until Hellmann‐Feynman forces fell below 0.01 eV Å^−1^ and total energy differences reached 10^−6^ eV using a 13 × 4 × 1 Monkhorst‐Pack k‐grid.^[^ ^61^ ^]^ Two collinear magnetic configurations were examined using FM Co layers as follows: (i) the FM state with uniform spin alignment, and (ii) the AFM state with antiparallel alignment between two Co layers. The IEC was quantified as *J* = Δ*E*/*A*, where Δ*E* = *E*_AFM_ − *E*_FM_, *E*_AFM_ and *E*_FM_ denotes the energy of AFM and FM configuration, and *A* denotes the interfacial area.^[^ ^34^ ^]^

## Conflict of Interest

The authors declare no conflict of interest.

## Supporting information



Supporting Information

## Data Availability

The data that support the findings of this study are available from the corresponding author upon reasonable request.
