# Supplementary material for: Electric Field Modulation of Interlayer Coupling via Piezostrain in a Synthetic Antiferromagnet
Source: Adv Sci (Weinh). 2025 Nov 28;13(8):e17798. doi: 10.1002/advs.202517798 (PMC12884806; doi:10.1002/advs.202517798)
Supplement: Supplementary file 1 — Supporting Information [file ADVS-13-e17798-s001.pdf]

Supporting Information

Electric Field Modulation of Interlayer Coupling via Piezostrain in a Synthetic Antiferromagnet

Yuichi Hisada*, Sachio Komori, Keiichiro Imura, Chenyu Shen, Yoshihiro Gohda, Calvin Ching Ian Ang, Wen Siang Lew, and Tomoyasu Taniyama**

Y. Hisada*, S. Komori, K. Imura, T. Taniyama**
Department of Physics, Nagoya University, Furo-cho, Chikusa-ku, Nagoya 464-8602, Japan
*Corresponding author's e-mail: * hisada.yuichi.t1@s.mail.nagoya-u.ac.jp
** taniyama.tomo@nagoya-u.jp

C. Shen, Y. Gohda
Department of Materials Science and Engineering, Institute of Science Tokyo, Yokohama 226-8501, Japan

C. C. I. Ang, W. S. Lew
School of Physical and Mathematical Sciences, Nanyang Technological University, 21 Nanyang Link, Singapore 637371, Singapore

Supplementary Note 1
Estimation of uniaxial magnetic anisotropy constant

We use a theoretical model based on the extended Stoner-Wohlfarth theory[1] to calculate the uniaxial magnetic anisotropy constant [K_{u1} (J m^{-3})] in Ru (3 nm)/Co (4 nm)/Ru (t_{Ru})/Co (3 nm)/Ru (20 nm)/PMN-PT(011) structures. The total energy (E) per unit volume in a synthetic antiferromagnet (SAF) with two ferromagnetic (FM) layers can be expressed by the following equation:

$$E\,(\theta_1, \theta_2) = K_{u1}\left(t_1\sin^2\theta_1 + t_2\sin^2\theta_2\right) - J\cos(\theta_1 - \theta_2) - \mu_0 MH\,[t_1\cos(\varphi - \theta_1) + t_2\cos(\varphi - \theta_2)], \quad \text{(S1)}$$

where K_{u1} is the uniaxial magnetic anisotropy constant, J is the interlayer exchange coupling (IEC) constant, M is the magnetization of the FM layers, $\mu_0 H$ is the external magnetic field, t_i (i = 1, 2) is the thickness of each FM layer, and θ_i (i = 1, 2) and φ are the angles of magnetization of each FM layer and the angle of the external magnetic field from the easy axis, respectively. Note that we have neglected the biquadratic coupling term, i.e., the magnetizations of the two FM layers are coupled by 90 degrees[2], and for simplicity, we have assumed the same value of K_{u1} for the Co (3 nm) and Co (4 nm) layers. We define $\varepsilon = E/M$, $H_k = 2K_{u1}/M$, and $H_1 = 2J/M$ and transform the variables (θ_1, θ_2) to (ξ, η), where $\xi = \theta_2 - \theta_1$, $\eta = (\theta_1 + \theta_2)/2$. Therefore, Eq. (S1) can be rewritten as Eq. (S2).

$$\varepsilon = \frac{H_k}{2}\left\{(t_1 + t_2)\left(\sin^2\eta\cos^2\left(\frac{\xi}{2}\right) + \cos^2\eta\sin^2\left(\frac{\xi}{2}\right)\right) - \frac{(t_1 - t_2)}{2}\sin2\eta\sin\xi\right\},$$
$$-\frac{H_1}{2}\cos\xi - \mu_0 H\left\{(t_1 + t_2)\cos\left(\frac{\xi}{2}\right)\cos(\eta-\varphi) + (t_1 - t_2)\sin\left(\frac{\xi}{2}\right)\sin(\eta-\varphi)\right\}. \quad (S2)$$

The equilibrium conditions can be described by the following equation:

$$\frac{\partial\varepsilon}{\partial\xi} = 0, \qquad \frac{\partial\varepsilon^2}{\partial\xi^2} \geq 0. \quad \text{(S3)}$$

Now, we consider the case where the external magnetic field is applied along the hard axis, i.e., when φ = π/2, and η = π/2. Thus, we obtain a simple relationship between $\mu_0 H_k$ and the saturation magnetic field ($\mu_0 H_s$) along the hard axis when $\xi = 0$, i.e., $\theta_1 = \theta_2$.

$$\mu_0 H_k = \mu_0 H_s + \frac{4J}{(t_1 + t_2)M_s}, \quad \text{(S4)}$$

where M_s is the saturation magnetization.

Figure S1(a) shows a schematic illustration of the Ru(3 nm)/Co(4 nm)/Ru(t_{Ru})/Co(3 nm)/Ru(20 nm)/PMN-PT(011) structure. Figure S1(b) shows the polar plot of the remanent magnetization (M_r) normalized by M_s as a function of the direction of the external magnetic field for the sample with t_{Ru} = 1.3 nm, indicating that the hard axis is along the PMN-PT [100] direction. Figure S1(c) shows the *M-H* curve along the hard axis for the sample with t_{Ru} = 1.3 nm. The value of $\mu_0 H_s$ is 617 ± 4.31 (mT), and J is approximately -0.152 (mJ m^{-2}) which can be deduced from Eq. (S6) [see Supplementary Note 2]. Therefore, we can extract the value of K_{u1} for the sample with t_{Ru} = 1.3 nm to be (3.15 ± 0.03) × 10^5 (J m^{-3}). The other samples also have the same order of magnitude for K_{u1} as this sample. As mentioned in the main text, the value of K_{u1} is much larger than $-J/t$ (where t is the thickness of the FM layer), since our samples with antiferromagnetic (AFM) coupling exhibit a metamagnetic transition[3,4]. Given J = -0.152 (mJ m^{-2}) and $t = (3 + 4)/2 = 3.5$ (nm), the value of $-J/t$ is approximately 4.34 × 10^4 (J m^{-3}), which is one order of magnitude smaller than K_{u1}. This large uniaxial magnetic anisotropy therefore induces the metamagnetic transition in our Co/Ru/Co SAFs.

Supplementary Note 2
How to evaluate the value of *J*

In SAFs, where the energy scale of uniaxial magnetic anisotropy is much smaller than that of the IEC, the value of $\mu_0 H_s$ in major hysteresis loops is proportional to J[3,4]. However, as discussed in Supplementary Note 1, our samples have a large K_{u1} value, indicating that $\mu_0 H_s$ is not an effective measure of J. Koplak et al. proposed a static magnetization switching diagram for SAFs with perpendicular magnetic anisotropy[5] and suggested that the switching magnetic fields $\mu_0 H_{c1}$, $\mu_0 H_{c2}$, and $\mu_0 H_{c3}$ [see Figure 3(a) in the main text] could be described by the following equation[5]:

$$\mu_0 H_{c1} = \frac{-2SJ - E_{2\mathrm{eff}}}{2m_2}, \tag{S5a}$$

$$\mu_0 H_{c2} = \frac{-2SJ + E_{1\mathrm{eff}}}{2m_1}, \tag{S5b}$$

$$\mu_0 H_{c3} = \frac{-2SJ + E_{2\mathrm{eff}}}{2m_2}. \tag{S5c}$$

where m_i is the magnetic moment of each FM layer, S is the surface area of the samples and $E_{i,\mathrm{eff}}$ is the energy barrier that blocks magnetization switching and is related to the magnetic anisotropy. The label i (i = 1, 2) corresponds to the FM layers of Co (4 nm) and Co (3 nm), respectively. From Eq. (S5a) and (S5c), we can derive the relationship between the switching magnetic fields and J:

$$\mu_0 (H_{c3} + H_{c1}) = \frac{-2SJ}{m_2}. \tag{S6}$$

The thicknesses of the two Co FM layers are different: 3 nm and 4 nm. Assuming precise control of the FM layer thickness in our deposition process, the value of m_2 is obtained from the value of the total magnetic moment (m_{tot}) measured by a vibrating sample magnetometer where $m_{\mathrm{tot}} = M_s t_{\mathrm{FM}} \mathrm{S}$ (t_{FM} is the total thickness of two Co layers) [see Figure 2 in the main text], i.e., $m_2 = (3/7)\ m_{\mathrm{tot}}$. Therefore, we can evaluate the value of J using the magnetic switching fields extracted from minor hysteresis loops.

Supplementary Note 3
Electric field modulation of $\mu_0(H_{c3} + H_{c1})$

Figures S2(a) to S2(e) show $\mu_0(H_{c3} + H_{c1})$ as a function of the electric field applied (E) to the samples with t_{Ru} = 1.2 nm, 1.25 nm, 1.3 nm, 1.32 nm and 1.34 nm, respectively. As can be seen in Figure S2, the value of $\mu_0(H_{c3} + H_{c1})$ sharply changes at around E = -0.04 MV m^{-1} to -0.20 MV m^{-1} as the electric field strength decreases from +0.6 MV m^{-1}. These values represent the reversal of electric polarization in PMN-PT substrates, indicating that inverse piezoelectric strain induces modulation of IEC strength.

Supplementary Note 4
In-plane XRD measurements under electric fields

Figures S3(a) and S3(c) show the in-plane XRD diffraction patterns measured around the PMN-PT(100) and (011) diffraction spots at different electric field strengths [these data were fitted using a Gaussian function, as shown in Figures S3(b) and S3(d)]. It can clearly be seen that the Bragg peak of both the (100) and the $(01\bar{1})$ plane shifts towards a lower angle as the electric field decreases from +0.6 MV m^{-1} to -0.6 MV m^{-1}.

Supplementary Note 5
Piezostrain behavior in the sample with t_{Ru} = 1.2 and 1.3 nm.

Figure S4 shows $\mu_0(H_{c3} + H_{c1})$ [blue curves] and the lattice constant for both [100] ($a_{[100]}$) and [01$\bar{1}$] ($a_{[01\bar{1}]}$) in PMN-PT(011) [red curves] as a function of applied electric field, swept from +0.6 to -0.6 MV/m. For the sample with t_{Ru} = 1.2 nm [(a) and (b)], the electric field dependence of $a_{[01\bar{1}]}$ aligns well with the IEC response, while for the sample with t_{Ru} = 1.3 nm [(c) and (d)], the electric field behavior of $a_{[100]}$ is in good agreement with the IEC response. These data indicates that the strain along [100] ([01$\bar{1}$]) is dominant for the IEC modulation in the sample with t_{Ru} = 1.2 nm (1.3 nm). Although the predominant strain direction contributing to the IEC modulation is different for the two samples, which is likely due to a structural phase transition [discussed in the supplementary note 6], Figure S4(b) and (c) clearly show that a tensile strain at the polarization reversal in PMN-PT plays a crucial role for enhancing the AFM IEC.

Supplementary Note 6
Reciprocal space mapping for the sample showing butterfly-like and hysteresis-like IEC responses

As mentioned in the main text and Supplementary Note 5, piezostrain behaviors in IEC exhibit two types of electric field responses: butterfly (t_{Ru} = 1.2 and 1.25 nm) and hysteresis (t_{Ru} = 1.3, 1.32, and 1.34 nm), and the direction of a tensile strain that critically enhances the IEC is different ([100] for t_{Ru} = 1.2 nm and [01$\bar{1}$] for t_{Ru} = 1.3 nm). To clarify the distinct behavior, we performed x-ray reciprocal space mapping (RSM) measurements on the samples showing butterfly-like and hysteresis-like IEC responses. Figure S5 shows the RSM data for the samples exhibiting butterfly-like (a)-(e) and hysteresis-like IEC responses (f)-(j) under electric fields swept from 0.6 to -0.6 MV/m, following the same protocol used for $\mu_0(H_{c1} + H_{c3})$ vs. E [Figure S5(a) and (f)]. RSM data were taken around the Bragg peak originating from the PMN-PT(022) plane. In the sample showing butterfly-like IEC behavior, although slight changes in the shape of the Bragg peak were observed under electric fields between −0.08 and −0.16 MV/m, corresponding to polarization reversal, the peak remained as a single spot. These single spots originate from the polarization vectors pointing toward r1+/r2+ in the positive E and r1-/r2- in the negative E [Figure 4(a) in the main text]. This indicates that ferroelectric (180°) domain switching is dominant. On the other hand, the sample exhibiting hysteresis-like IEC response shows clear peaks splitting into two components when the electric field was swept from −0.16 to −0.20 MV/m [Figure S5(h) and (i)]. Upon further decreasing the electric field down to -0.6 MV/m, the split patterns are more noticeable (red dotted circle). Since the value of $Q_z/2\pi$ in the central part of the Bragg peak for the sample with hysteresis-like IEC behavior is almost identical for the single peak for the sample with butterfly IEC behavior, the lower part of the Bragg peak cannot be solely explained by the polarization domain switching with 71°, 109°, and 180° pathways. In other words, the lower part of the splitting peak does not originate from ideal domain switching in PMN-PT. We attribute this irregular peak splitting to a structural phase transition in the PMN-PT substrates, which are near the morphotropic phase boundary and often cause unexpected strain behavior[6,7]. However, further investigation into the origin

of this complicated strain behavior is necessary for the application of efficient spintronic devices based on artificial multiferroics.

Supplementary Note 7
Electric-field modulation behavior of $\mu_0 H_{c1}$ and $\mu_0 H_{c3}$

Figures S6(a)–(j) show the minor hysteresis loops in the vicinity of $\mu_0 H_{c1}$ [(a) – (e)] and $\mu_0 H_{c3}$ [(f) – (j)] under electric fields in the sample with t_{Ru} = 1.2 nm, 1.25 nm, 1.3 nm, 1.32 nm and 1.34 nm, respectively. Notably, the electric field modulation behavior of $\mu_0 H_{c1}$ and $\mu_0 H_{c3}$ depends on t_{Ru}, i.e., the IEC strength. In samples with relatively strong AFM coupling (t_{Ru} = 1.2 and 1.25 nm), both $\mu_0 H_{c1}$ and $\mu_0 H_{c3}$ shift towards the high magnetic field region when an electric field is applied. In samples with weak AFM coupling, on the other hand, $\mu_0 H_{c1}$ shifts towards the high magnetic field region, while $\mu_0 H_{c3}$ shifts towards the low magnetic field region.

Supplementary Note 8
Estimation of electric field modulation of uniaxial magnetic anisotropy energy

We can also estimate the value of $E_{i,\mathrm{eff}}$ (i = 1, 2), which is dependent on the electric field and related to the uniaxial magnetic anisotropy energy in both Co layers, using Eq. (S5a) - (S5c) and (S6) in Supplementary Note 2. The magnetic anisotropy energy of the 3-nm and 4-nm Co layers per volume [i.e., $E_{2\mathrm{eff}}/V_{\mathrm{Co\,(3\,nm)}}$ and $E_{1\mathrm{eff}}/V_{\mathrm{Co\,(4\,nm)}}$, respectively] can be obtained from Eq. (S5a)–(S5c) and (S6):

$$\frac{E_{2\mathrm{eff}}}{V_{\mathrm{Co\,(3\,nm)}}} = \frac{m_2 \cdot \mu_0\,(H_{c3} - H_{c1})}{V_{\mathrm{Co\,(3\,nm)}}}, \tag{S7}$$

$$\frac{E_{1\mathrm{eff}}}{V_{\mathrm{Co\,(4\,nm)}}} = \frac{2m_1 \cdot \mu_0 H_{c2} - m_2 \cdot \mu_0\,(H_{c3} + H_{c1})}{V_{\mathrm{Co\,(4\,nm)}}}, \tag{S8}$$

where $V_{\mathrm{Co\,(3\,or\,4\,nm)}}$ is the volume of the 3 or 4 nm Co layer, and $\mu_0 H_{c2}$ corresponds to the saturation magnetic field ($\mu_0 H_s$) value in the major hysteresis loop. Figures S7 and S8 show $E_{1\mathrm{eff}}$ and $E_{2\mathrm{eff}}$ as a function of E, respectively. In almost all samples, $E_{1\mathrm{eff}}$ [for the Co (4 nm) layer] increases sharply while $E_{2\mathrm{eff}}$ [for the Co (3 nm) layer] decreases when the electric polarization switches from in-plane to out-of-plane in PMN-PT(011). Conversely, our micromagnetic simulations show that the balance between the enhancement of AFM coupling induced by the electric field and the reduction of uniaxial magnetic anisotropy energy determines the behavior of the minor hysteresis loops of our samples, as shown in Figure 5. The estimation of the electric field-dependent magnetic anisotropy for the 4-nm Co layer obtained from this experiment is inconsistent with the simulation results. However, micromagnetic simulations show that the modulation of $E_{1\mathrm{eff}}$ has a smaller influence on the behavior of the minor hysteresis loop than $E_{2\mathrm{eff}}$, as shown in Figure S9(d) in Supplementary Note 9, which will be discussed later. This suggests that the reduction of magnetic anisotropy energy of the 3 nm Co layer, rather than the 4 nm Co layer, is one of the dominant factors in the electric field modulation of the minor hysteresis loop for our samples. Therefore, the relationship between the modulation of the IEC by an electric field and the magnetic anisotropy

energy, as obtained from micromagnetic simulations, is in qualitative agreement with the experimental results.

Supplementary Note 9
Micromagnetic simulations under various conditions

Figures S9(a)–(d) show the amount of change in the switching magnetic fields $\mu_0 H_{c1}$ and $\mu_0 H_{c3}$ under various conditions, as determined by micromagnetic simulations. Changing the value of J causes both $\mu_0 H_{c1}$ and $\mu_0 H_{c3}$ to shift towards the high magnetic field region. Conversely, $\mu_0 H_{c1}$ ($\mu_0 H_{c3}$) shifts towards the high (low) magnetic field region when the magnetic anisotropy energy of any Co layer(s) is reduced [Figure S9(b)–(d)]. Notably, when the magnetic anisotropy energy of only the Co (4 nm) layer changes, $\mu_0 H_{c1}$ and $\mu_0 H_{c3}$ show little change compared to the other cases, as shown in Figure S9(d) and (h). This indicates that enhancing J and/or reducing the magnetic anisotropy energy of the Co (3 nm) layer are the dominant factors in modulating the minor hysteresis loop in our SAFs.

Supplementary Note 10
Atomic configurations of interfaces between hcp Co and hcp Ru $(10\bar{1}3)$

Bulk hexagonal close-packed (hcp) Co exhibits lattice constants of a = 2.48 Å (in-plane) and c = 4.02 Å (out-of-plane), while bulk hcp Ru displays corresponding parameters of a = 2.72 Å and c = 4.29 Å, as determined by first-principles calculations on the basis of density functional theory (DFT) as shown in Figure S10(a). Low–index epitaxial relationships between hexagonal Co and Ru were enumerated with the Substrate-Analyzer class in pymatgen[8], which implements the coincidence-lattice algorithm of Zur and McGill[9]. All symmetrically distinct surface terminations satisfying |h|, |k|, |l|≤3 for both film and substrate were generated, after which a reduced 2 × 2 coincidence lattice was constructed for every pair and its in-plane Lagrangian strain tensor ε was evaluated. Candidate matches were ranked by the scalar von Mises invariant $\varepsilon_{vm} = [(\varepsilon_{11} - \varepsilon_{22})^2 + 6{\varepsilon_{12}}^2]^{1/2}/\sqrt{2}$, which correlates with elastic energy density while treating tension and compression symmetrically. Restricting the substrate orientation to Ru $(10\bar{1}3)$ and retaining only the global minimum in ε_{vm} yielded the orientation relationship Co $(20\bar{2}1)$∥Ru $(10\bar{1}3)$ with ε_{vm} = 0.54 %, which was adopted for modeling the heterostructure as shown in Figure S10(b).

References

[1]. H. Fujiwara, and M. R. Parker, "Analytical model of giant MR in multilayers with biquadratic coupling," *Journal of Magnetism and Magnetic Materials* **135**, (1994): L23. https://doi.org/10.1016/0304-8853(94)90169-4

[2]. S. O. Demokritov, "Biquadratic interlayer coupling in layered magnetic systems," *Journal of Physics D: Applied Physic* **31**, (1998): 925. https://doi.org/10.1088/0022-3727/31/8/003

[3]. B. Dieny, J. P. Gavigan, and J. P. Rebouillat, "Magnetisation processes, hysteresis and finite-size effects in model multilayer systems of cubic or uniaxial anisotropy with

antiferromagnetic coupling between adjacent ferromagnetic layers," *Journal of Physics: Condensed Matter* **2**, (1990): 159. https://doi.org/10.1088/0953-8984/2/1/013

[4]. P. J. H. Bloemen, H. W. van Kesteren, H. J. M. Swagten, and W. J. M. de Jonge, "Oscillatory interlayer exchange coupling in Co/Ru multilayers and bilayers," *Physical Review B* **50**, (1994): 13505. https://doi.org/10.1103/PhysRevB.50.13505

[5]. O. Koplak, A. Talantsev, Y. Lu, et al., "Magnetization switching diagram of a perpendicular synthetic ferrimagnet CoFeB/Ta/CoFeB bilayer," *Journal of Magnetism and Magnetic Materials* **433**, (2017): 91. https://doi.org/10.1016/j.jmmm.2017.02.047

[6]. D. Viehland, and J. F. Li, "Anhysteretic field-induced rhombhohedral to orthorhombic transformation in 〈110〉 -oriented $0.7Pb(Mg_{1/3}Nb_{2/3})O_3$-$0.3PbTiO_3$ crystals," *Journal of Applied Physics* **92**, (2002): 7690. https://doi.org/10.1063/1.1524016

[7]. H. Cao, F. Bai, N. Wang, et al., "Intermediate ferroelectric orthorhombic and monoclinic MB phases in [110] electric-field-cooled $Pb(Mg_{1/3}Nb_{2/3})O_3$-30%$PbTiO_3$ crystals," *Physical Review B* **72**, (2005): 064104. https://doi.org/10.1103/PhysRevB.72.064104

[8]. S. P. Ong, W. D. Richards, A. Jain, et al., "Python Materials Genomics (pymatgen): a robust, open-source Python library for materials analysis," *Computational Materials Science* **68**, (2013): 314. https://doi.org/10.1016/j.commatsci.2012.10.028

[9]. A. Zur, and T. C. McGill, "Lattice match: an application to heteroepitaxy," *Journal of Applied Physics* **55**, (1984): 378. https://doi.org/10.1063/1.333084

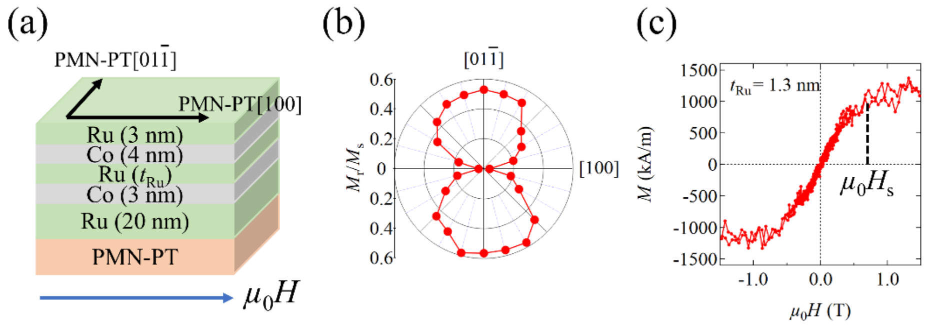

Figure S1 (a) A schematic illustration of Ru (3 nm) /Co (4 nm) /Ru (t_{Ru}) /Co 3 nm) /Ru (20 nm) /PMN-PT(011) structures. (b) The polar plot of the remanent magnetization (M_r) normalized by saturation magnetization (M_s) as a function of external magnetic field direction. (c) The magnetization curves along the hard axis (PMN-PT[100]).

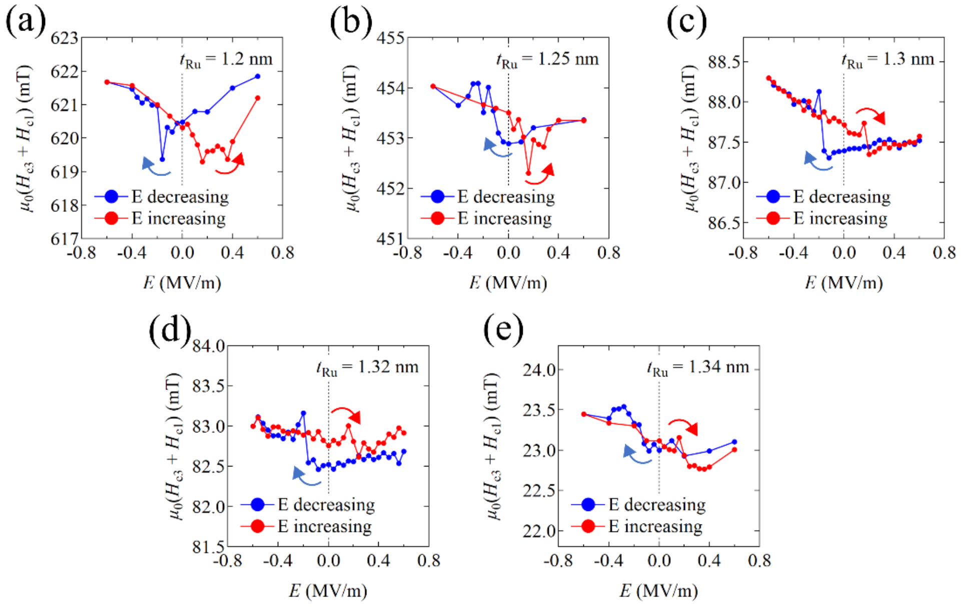

Figure S2 (a)-(e) Electric field modulation of $\mu_0(H_{c3}+H_{c1})$ values for the sample with t_{Ru} = 1.2 nm, 1.25 nm, 1.3 nm, 1.32 nm, and 1.34 nm, respectively. The red (blue) curve corresponds to the increasing (decreasing) electric field sequence.

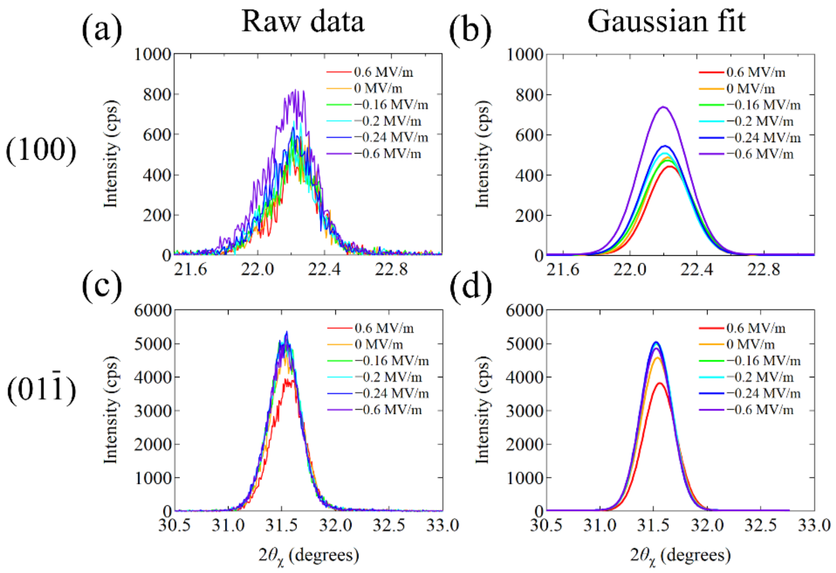

Figure S3 In-plane XRD diffraction patterns under electric fields measured around PMN-PT(100) [(a) and (b)] and $(01\bar{1})$ [(c) and (d)] diffraction spot. The raw data [(a) and (c)] are fitted by the Gaussian function [(b) and (d)].

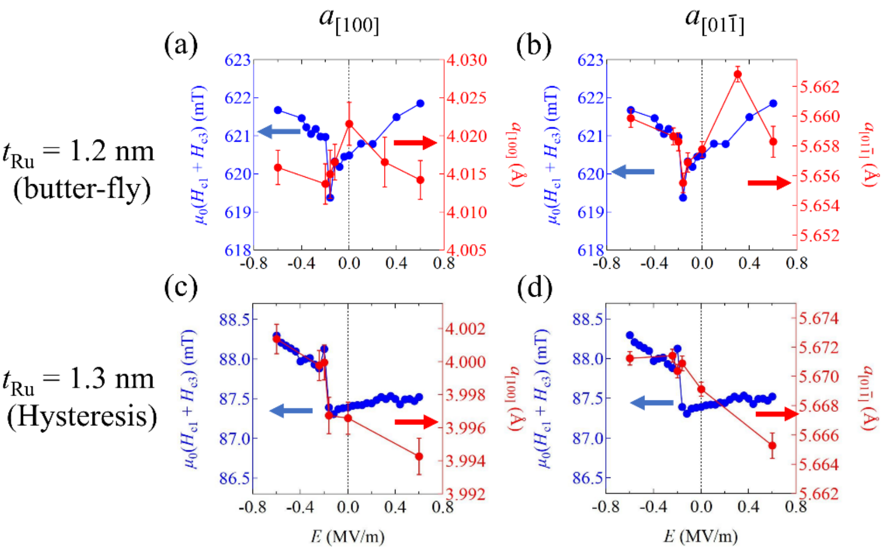

Figure S4 $\mu_0(H_{c3+}H_{c1})$ [blue data] and the lattice constant for both [$01\bar{1}$] ($a_{[100]}$) and [$01\bar{1}$] ($a_{[01\bar{1}]}$) in PMN-PT(011) [red data] as a function of applied electric field swept from +0.6 to -0.6 MV/m. (a) and (b) [(c) and (d)] are the data for the sample with t_{Ru} = 1.2 nm (t_{Ru} = 1.3 nm) for both $a_{[100]}$ (a) [(c)] and $a_{[01\bar{1}]}$ (b) [(d)], respectively.

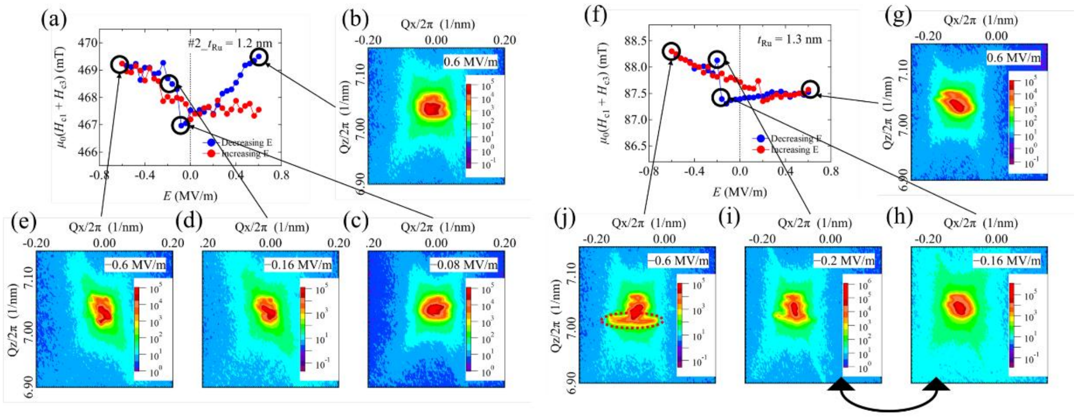

Figure S5 (a) and (f) $\mu_0(H_{c1} + H_{c3})$ as a function E for the sample with a butterfly-like IEC response (t_{Ru} = 1.2 nm) and a hysteresis-like IEC response (t_{Ru} = 1.3 nm), respectively. (b)-(e), and (g)-(j) RSM data for the samples with t_{Ru} = 1.2 nm and t_{Ru} = 1.3 nm under the electric fields corresponding to the electric field sweeping protocol as shown in Figure S5(a) and (f), respectively. Note that the Ru thickness of the sample exhibiting the butterfly IEC response is different sample from the sample shown in Figure S2 (a), but the IEC response behaves hysteretic similarly.

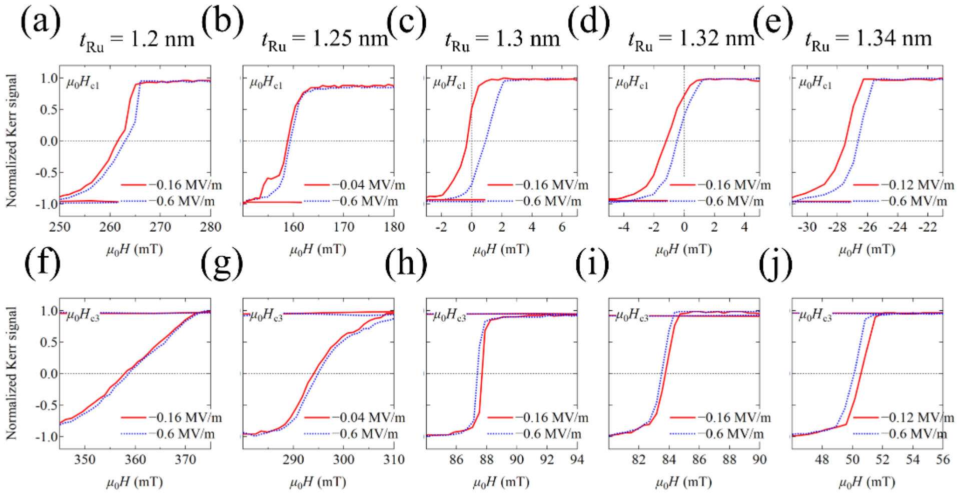

Figure S6 Electric field modulation of the magnetization curves in the vicinity of μ_0H_{c1} and μ_0H_{c3} for the samples with t_{Ru} = 1.2 nm, 1.25 nm, 1.3 nm, 1.32 nm, and 1.34 nm, respectively. The red curves and blue dots represent the data under E = −0.16 MV m^{-1} and −0.6 MV m^{-1}, respectively.

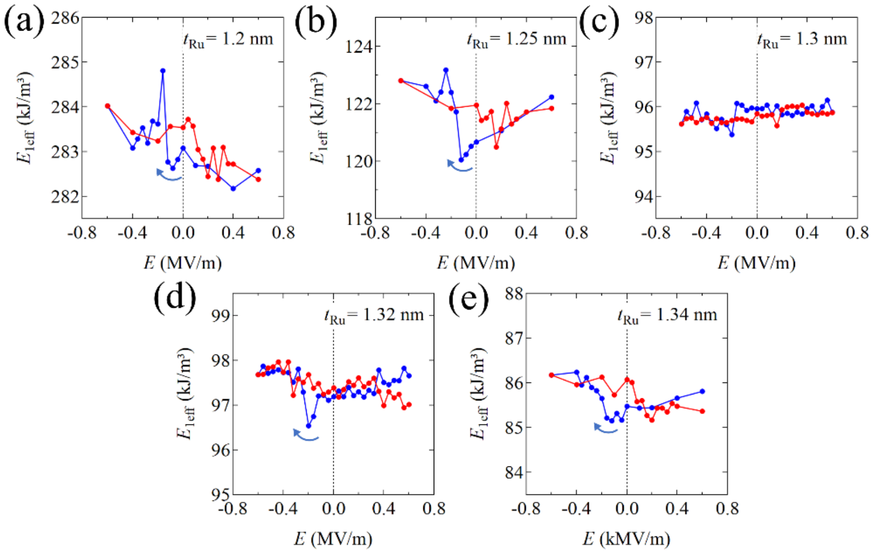

Figure S7 (a)-(e) Magnetic anisotropy energy $E_{1\mathrm{eff}}$ as a function of E for the samples with t_{Ru} = 1.2, 1.25, 1.3, 1.32, and 1.34 nm, respectively. The red (blue) curve corresponds to the increasing (decreasing) electric field sequence.

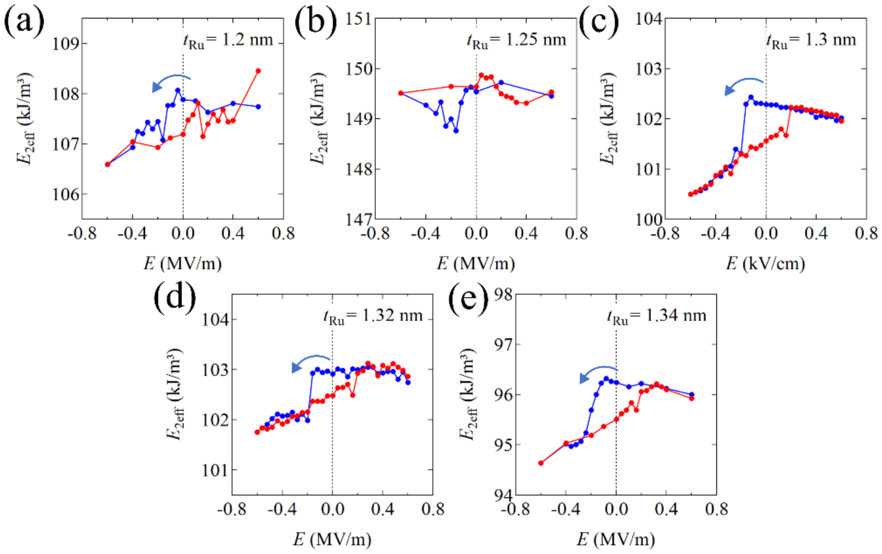

Figure S8 (a)-(e) Magnetic anisotropy energy E_{2eff} as a function of E for the samples with t_{Ru} = 1.2, 1.25, 1.3, 1.32, and 1.34 nm, respectively. The red (blue) curve corresponds to the increasing (decreasing) electric field sequence.

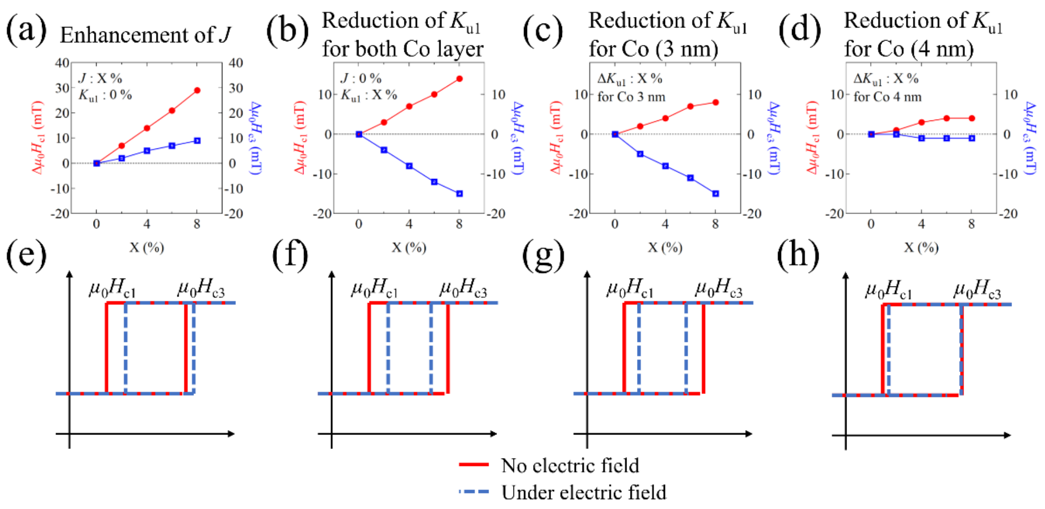

Figure S9 (a)-(d) Micromagnetic simulation results of the amount of change in the switching magnetic field ($\mu_0 H_{c1}$ and $\mu_0 H_{c3}$) under various conditions: (a) under enhancement of J, (b)-(d) under reduction of the magnetic anisotropy energy of both Co layer, Co (3 nm) layer, and Co (4 nm) layer, respectively. (e)-(h) Schematic illustrations of minor hysteresis loop corresponding to the micromagnetic simulation results of (a)-(d), respectively.

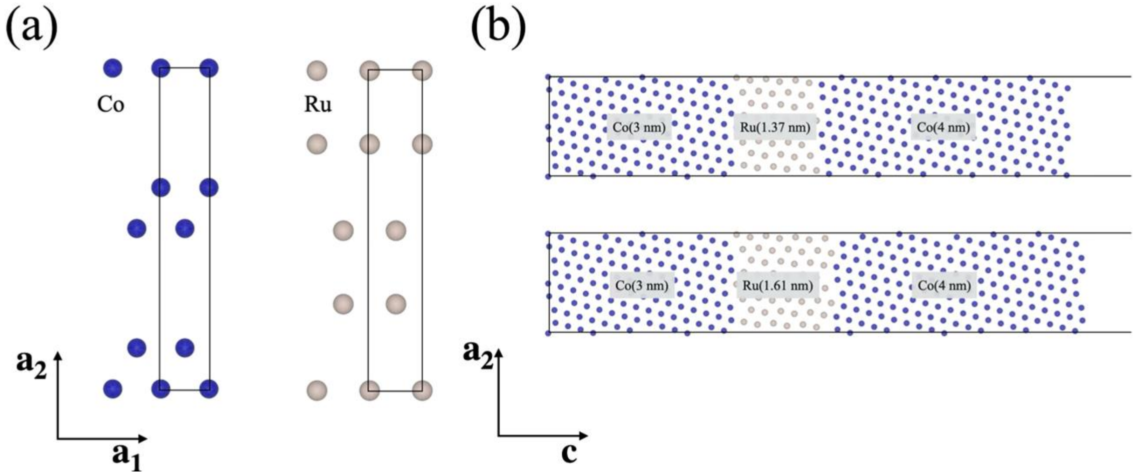

Figure S10 (a) Top view of the Co $(20\bar{2}1)$ plane and Ru $(10\bar{1}3)$ plane; (b) Side view along the $\mathbf{a_1}$ axis, showing the layer sequences from top to bottom: Co (3 nm)/Ru (1.37 nm)/Co (4 nm) and Co (3 nm)/Ru (1.61 nm)/Co (4 nm) heterostructure.
